# Prognostic significance of lncRNA DANCR expression in human cancers: a systematic review and meta-analysis

**DOI:** 10.1042/BSR20181627

**Published:** 2021-08-12

**Authors:** Wei Liu, Qin-Peng Wang, Jia Guo

**Affiliations:** Department of Neurology, Lanzhou University Second Hospital, No. 82 Cuiyingmen, Lanzhou 730030, Gansu province, P.R. China

**Keywords:** Cancer, LncRNA DANCR, Meta-analysis, Prognosis

## Abstract

Several studies demonstrated that lncRNA differentiation antagonizing non-protein coding RNA (lncRNA DANCR) expression might have the potential capacity to predict the cancer prognosis; however, definite conclusion has not been obtained. The aim of this meta-analysis was to evaluate the prognostic value of lncRNA DANCR expression in cancers. PubMed, Web of Science, Scopus, and Embase were comprehensively searched for relevant studies. Studies meeting all inclusion standards were included into this meta-analysis. The analysis of overall survival (OS), disease-free survival (DFS), or clinicopathological features was conducted. Total 11 studies containing 1154 cancer patients were analyzed in this meta-analysis. The results showed, compared with low lncRNA DANCR expression, high lncRNA DANCR expression was significantly associated with shorter OS (hazard ratio [HR] = 1.85; 95% CI = 1.52–2.26; *P*<0.01) and DFS (HR = 1.82; 95% CI = 1.43–2.32; *P*<0.01) in cancers. Besides, high lncRNA DANCR expression predicted deeper tumor invasion (*P*<0.01), earlier lymph node metastasis (*P*<0.01), earlier distant metastasis (*P*<0.01), and more advanced clinical stage (*P*<0.01) compared with low lncRNA DANCR expression in cancer populations. High lncRNA DANCR expression was associated with worse prognosis compared with low lncRNA DANCR expression in cancers. LncRNA DANCR expression could serve as a prognostic factor of human cancers.

## Introduction

Cancer has become a crucial public health problem and a leading cause of death worldwide [1,2]. Despite of tremendous improvement of diagnosis and treatments, the prognosis of many cancer patients at terminal stage remains disappointing [2,3]. The lack of efficient biomarkers to serve as treatment targets and predict the prognosis is considered as the main reason for this dilemma. Therefore, a growing number of researchers begin to look for optimal biomarkers of human cancers [4,5].

With the rapid development of high-throughput sequencing technology, increasing lncRNAs are discovered and have become the research hotspots [6]. LncRNA, greater than 200 nts in length, is a major type of ncRNAs without protein-coding capability [7]. Recently, lncRNAs have been proved to be closely associated with tumorigenesis, differentiation, invasion, and metastasis of cancers [8,9]. LncRNA differentiation antagonizing non-protein coding RNA (lncRNA DANCR), a kind of lncRNA, is located on human chromosome 4 [10]. Recently, accumulating studies have supported a substantial role of lncRNA DANCR expression in the cancer prognosis [11–21]. However, conclusion has not been reached for the contradictory results amongst different publications [11–21]. Here, we conducted this systematic review and meta-analysis to determine the prognostic value of lncRNA DANCR expression in cancers.

## Materials and methods

The present study was performed in accordance with Preferred Reporting Items for Systematic Reviews and Meta-Analyses (PRISMA) [22] (Supplementary Table S1)

### Literature search and selection

PubMed, Web of Science, Scopus, and Embase were comprehensively searched up to 15 January 2019. The strategy was as following: (‘lncRNA differentiation antagonizing non-protein coding RNA’ OR ‘lncRNA DANCR’ OR ‘lncRNA DANCR’ OR ‘DANCR’) AND (‘cancer’ OR ‘tumor’ OR ‘neoplasm’ OR ‘carcinoma’). There was no restriction on the language. References of retrieved studies were also checked to avoid missing relevant studies. All studies were selected according to inclusion and exclusion criteria.

### Inclusion and exclusion criteria

The study was considered to be eligible if it satisfied the following criteria: (1) patients were pathologically diagnosed as cancers; (2) prognostic value of lncRNA DANCR expression in cancers was assessed; (3) overall survival (OS), disease-free survival (DFS), recurrence-free survival (RFS), or clinicopathological feature was reported; (4) patients were divided into two groups based on the expression level of lncRNA DANCR; (5) full text and sufficient data were provided. The following studies were excluded: reviews, comments, letters, case reports, cell experiments, animal experiments, unpublished studies, and duplications.

### Data extraction and quality evaluation

Data extraction and quality evaluation were independently operated by two authors. Any disagreement would be solved by discussing with the third author. The following items were extracted: first author, publication year, country, sample size, gender, expression level of lncRNA DANCR, cancer type, outcomes, and analysis model of OS. As for prognostic variables (e.g., OS, DFS, and RFS), HR and corresponding 95% CI were directly extracted from published studies or indirectly calculated from survival curves if only survival curves were available [23]. Moreover, if HR and 95% CI were simultaneously provided in the multivariate analysis and univariate analysis, the former were used. The analysis model of OS was considered as univariate analysis when HR and 95% CI were indirectly calculated from survival curves. Quality of included studies was assessed with Newcastle–Ottawa Scale (NOS). We considered studies with scores no less than six as high-quality studies [24].

### Statistical analysis

For prognostic variables, such as OS, DFS, and RFS, HR and 95% CI were pooled to assess the relationship between lncRNA DANCR expression and cancer prognosis. As for dichotomous, such as gender, lymph node metastasis, and clinical stage, odds ratio (OR) and 95% CI were applied to detect the overall effects. Heterogeneity was assessed using Cochran’s Q test and Higgins I-squared statistics. I^2^ > 50% and/or *P*<0.10 suggested obvious heterogeneity amongst studies, as a result, a random-effect model was utilized. Alternatively, a fixed-effect model was used. Sensitivity analysis was done by omission of each single study. Publication bias was evaluated using Begg’s test and funnel plots. All analyses were conducted by Reviewer Manager 5.3 (Cochrane Collaboration, Copenhagen, Denmark) and Stata 12.0 (Stata Corporation, College Station, Texas, U.S.A.). All *P* values were two sides and difference was considered significant when *P* value was less than 0.05.

## Results

### Literature search and selection

**Figure 1:**
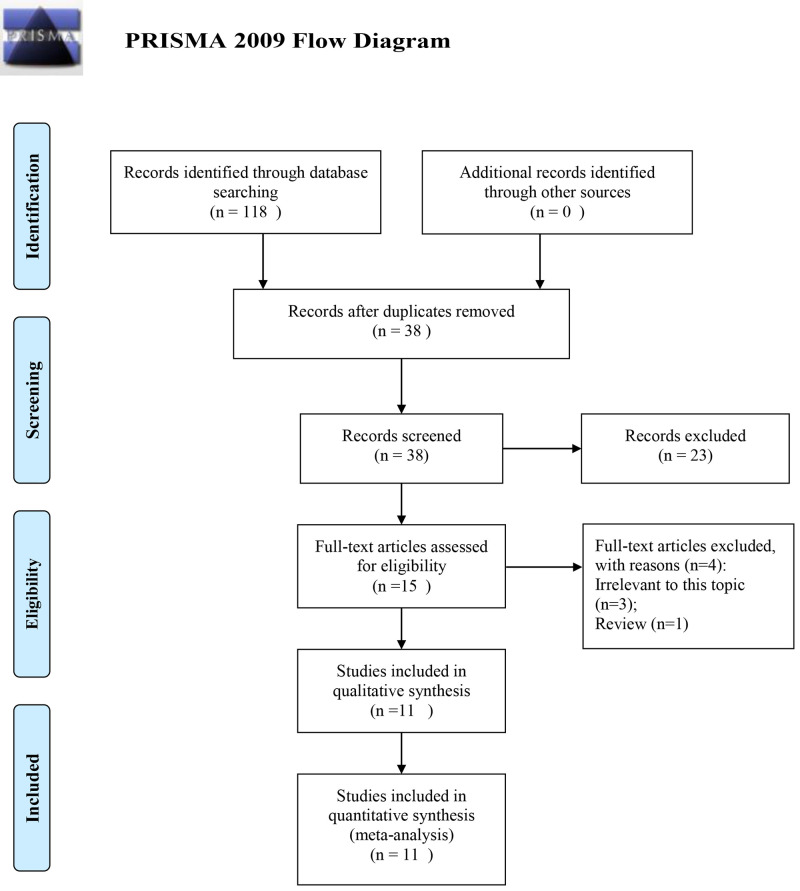
Flow chart of literature search and selection

A total of 118 articles were initially retrieved from four common databases (Figure 1). A total of 38 articles remained for further evaluation after the removal of duplicates. Then, 23 articles were directly excluded by scanning titles or abstracts. Regarding to the remaining 15 articles, four articles were excluded by evaluating full-texts. Ultimately, 11 studies were included for further analysis [11–21].

### Basic information of included studies

The basic information of studies included was listed in Table 1. A total of 11 studies containing 1154 cancer patients were included into this research [11–21]. Especially, Yuan et al. study consisted of two cohorts (cohort 1: Chinese population; cohort 2: Korea population) [21]; therefore, 12 cohorts were analyzed in this research. Seven studies reported the clinical stage of patients (I/II: 235 patients; III/IV: 317 patients) [11,13–16,18,19]. Besides, lncRNA DANCR expression in cancer tissues was evaluated using quantitative reverse transcription polymerase chain reaction (qRT-PCR) in all studies [11–21]. Three studies used the median value [13,14,21] and one study used the normalized value to divide patients into high or low lncRNA DANCR expression groups [16]; however, the other studies failed to provide the definite cut-off value [11,12,15,17–20]. Additionally, seven types of cancer were investigated, including gastric cancer [11,14,15], osteosarcoma [12,19], non-small cell lung cancer [17], colorectal cancer [13,18], breast cancer [16], and glioma [20] as well as hepatocellular carcinoma [21]. Patients received surgical treatment in eight studies [11–13,15,16,18–20]; nevertheless, treatment of patients in the other studies was not available [14,17,21]. Regarding to outcomes, nine studies reported clinicopathological parameters (CPs) [11–16,18–20], eight studies reported OS [11–13,16,17,19–21], two studies reported DFS [12,13], and one study reported RFS [21]. Moreover, OS was evaluated using multivariate analysis model in three cohorts [12,13,21] and univariate analysis model in six cohorts [11,16,17,19–21]. NOS score was larger than six in all studies, which indicated all studies were with high quality [11–21].

**Table 1:** Basic information of included studies

Study	Country	Sample size (n)	Clinical stage (I+II/III+IV)	Detection methods	Cut-off value	Cancer type	Treatments	Outcomes	Analysis model	NOS
Hao 2017 [11]	China	118	48/70	qRT-PCR	NA	Gastric cancer	Surgery	CP, OS	U	7
Jiang 2017 [12]	China	34	NA	qRT-PCR	NA	Osteosarcoma	Surgery	CP, DFS, OS	M	8
Jiang 2018 [17]	China	128	NA	qRT-PCR	NA	NSCLC	NA	OS	U	6
Liu 2015 [13]	China	104	37/67	qRT-PCR	Median	Colorectal cancer	Surgery	CP, DFS, OS	M	8
Mao 2017 [14]	China	60	33/27	qRT-PCR	Median	Gastric cancer	NA	CP	NA	6
Pan 2018 [15]	China	65	19/46	qRT-PCR	NA	Gastric cancer	Surgery	CP	NA	6
Sha 2017 [16]	China	63	37/26	qRT-PCR	≤0.5/≥2.0^†^	Breast cancer	Surgery	CP, OS	U	7
Wang 2018 [19]	China	95	42/53	qRT-PCR	NA	Osteosarcoma	Surgery	CP, OS	U	7
Yang 2018 [20]	China	82	NA	qRT-PCR	NA	Glioma	Surgery	CP, OS	U	7
Yuan 2016 [21]	China	135	NA	NA	Median	Hepatocellular carcinoma	NA	RFS, OS	M	7
Yuan 2016 [21]	Korea	223	NA	NA	Median	Hepatocellular carcinoma	NA	RFS, OS	U	6
Zeng 2018 [18]	China	47	19/28	qRT-PCR	NA	Colorectal cancer	Surgery	CP	NA	6

† The normalized values ≤0.5 and ≥2.0 were used to determine low-expression and high-expression of DANCR expression, respectively.

M, multivariate; NA, not available; NSCLC, non-small cell lung cancer; U, univariate.

### Meta-analysis for the association between lncRNA DANCR expression and prognosis

**Figure 2:**
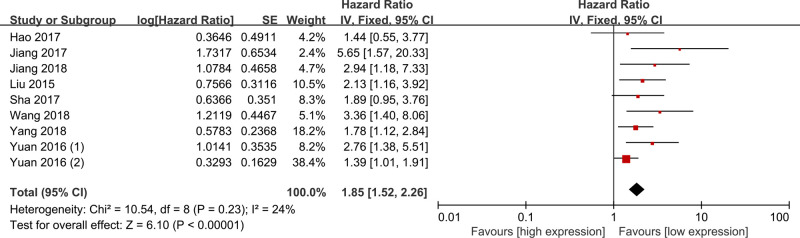
Meta-analysis of the association between lncRNA DANCR expression and OS

Eight studies evaluated the correlation between lncRNA DANCR expression and OS, and all of them were included into the analysis [11–13,16,17,19–21]. As shown in Figure 2, a fixed-effect model was used because there was no obvious heterogeneity amongst included studies (I^2^ = 24%, *P*=0.23). High lncRNA DANCR expression was significantly correlated with shorter OS compared with low lncRNA DANCR expression in cancers (HR = 1.85; 95% CI = 1.52–2.26; *P*<0.01).

To further explore the prognostic value of lncRNA DANCR expression in cancers, subgroup analysis was performed (Table 2). Significant relationship between high lncRNA DANCR expression and shorter OS was detected in all subgroup analyses (*P*<0.05).

**Table 2:** Subgroup analysis for the association between lncRNA DANCR expression and OS

Variables	Cohorts (n)	HR (95% CI)	*P* value	Heterogeneity		Model
				I^2^ (%)	*P* value	
**Analysis model**						
Multivariate	3	2.63 (1.71–4.05)	<0.01^‡^	0	0.4	Fixed
Univariate	6	1.69 (1.35–2.11)	<0.01^‡^	9	0.36	Fixed
**Sample size (n)**						
>100	5	1.71 (1.34–2.18)	<0.01^‡^	24	0.27	Fixed
≤100	4	2.16 (1.54–3.03)	<0.01^‡^	26	0.25	Fixed
**Cut-off value**						
Median	3	1.66 (1.28–2.15)	<0.01^‡^	49	0.14	Fixed
Others	6	2.14 (1.59–2.90)	<0.01^‡^	1	0.41	Fixed
**Treatments**						
Surgery	6	2.08 (1.56–2.76)	<0.01^‡^	0	0.47	Fixed
Others	3	2.01 (1.17–3.47)	0.01^‡^	58	0.09	Random
**Cancer type**						
Gastrointestinal cancers	4	1.64 (1.28–2.11)	<0.01^‡^	24	0.27	Fixed
Others	5	2.24 (1.63–3.08)	0.01^‡^	8	0.36	Fixed

‡ The association was considered significant when *P*<0.05.

**Figure 3:**
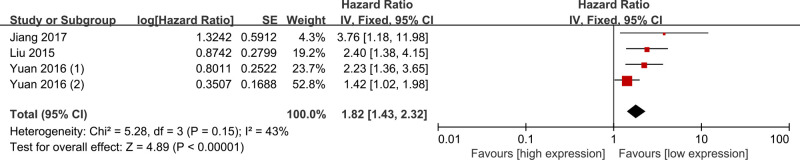
Meta-analysis of the association between lncRNA DANCR expression and DFS

Two studies reported DFS [12,13] and one study reported RFS [21], and all of them were included into the analysis for DFS (Figure 3). A fixed-effect model was used because of the moderate heterogeneity (I^2^ = 43%, *P*=0.15). Compared with patients with low lncRNA DANCR expression, patients with high lncRNA DANCR expression tended to have a shorter DFS (HR = 1.82; 95% CI = 1.43–2.32; *P*<0.01).

### Meta-analysis for the association between lncRNA DANCR expression and CPs

Meta-analyses for the association between lncRNA DANCR expression and CPs were conducted (Table 3). There was no obvious relationship between the expression level of lncRNA DANCR and age (*P*=0.26), gender (*P*=0.42), tumor size (*P*=0.59) or tumor differentiation (*P*=0.11). However, compared with low expression level of lncRNA DANCR, high expression level of lncRNA DANCR was significantly associated with deeper tumor invasion (*P*<0.01), earlier lymph node metastasis (*P*<0.01), earlier distant metastasis (*P*<0.01), and more advanced clinical stage (*P*<0.01).

**Table 3:** Meta-analysis for the association between lncRNA DANCR expression and CPs

Variables	Studies (n)	Patients (n)	OR (95% CI)	*P* value	Heterogeneity		Model
					I^2^ (%)	*P* value	
Age (old versus young)	7	467	1.25 (0.85–1.83)	0.26	0	0.81	Fixed
Gender (male versus female)	7	523	1.16 (0.81–1.67)	0.42	40	0.13	Fixed
Tumor size (large versus small)	7	539	1.31 (0.50–3.46)	0.59	84	<0.01	Random
Tumor differentiation (poor versus well)	5	394	1.99 (0.85–4.70)	0.11	73	<0.01	Random
Invasion depth (T3/T4 versus T1/T2)	3	216	2.68 (1.43–5.04)	<0.01‡	0	0.41	Fixed
Lymph nodes metastasis (yes versus no)	5	339	5.49 (3.29–9.16)	<0.01‡	0	0.67	Fixed
Distant metastasis (yes versus no)	3	207	4.75 (2.17–10.41)	<0.01‡	0	0.72	Fixed
Clinical stage (III/IV versus I/II)	6	435	4.11 (2.68–6.31)	<0.01‡	0	0.94	Fixed

‡ The association was considered significant when *P*<0.05.

### Publication analysis and sensitivity analysis

**Figure 4:**
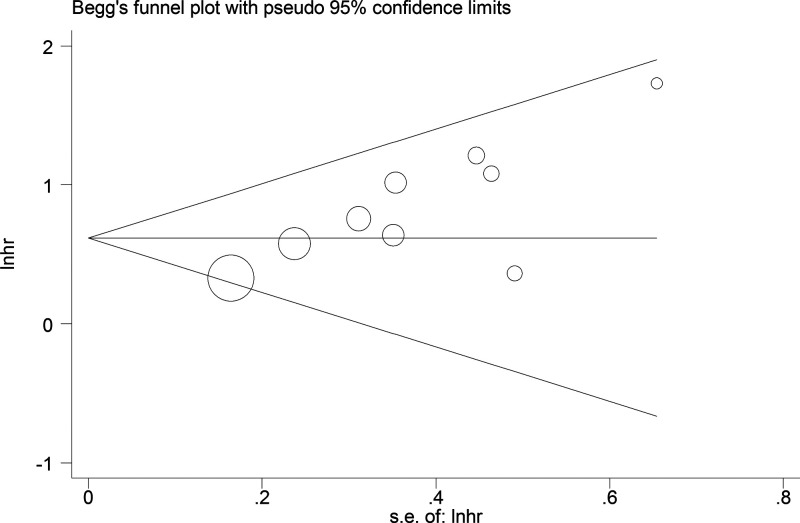
Begg’s test for meta-analysis of the association between lncRNA DANCR expression and OS

**Figure 5:**
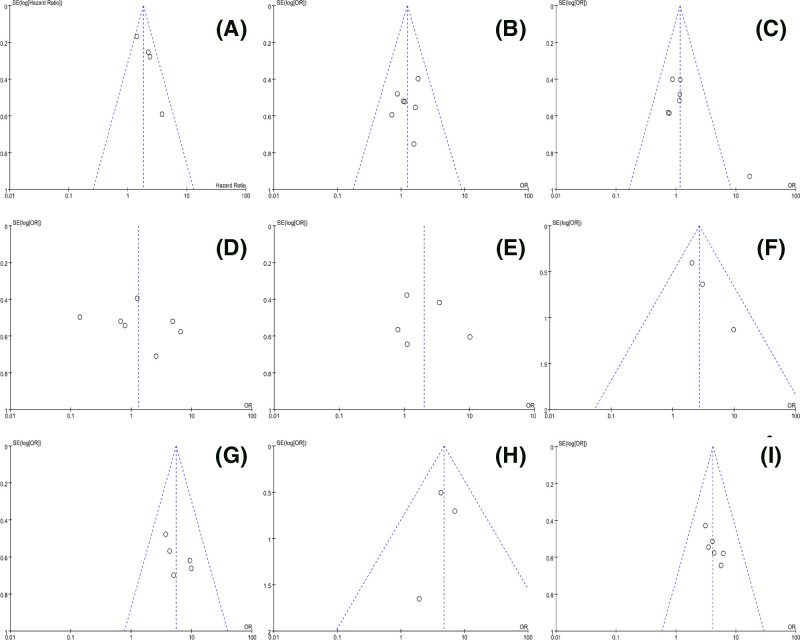
Funnel plots for the meta-analyses of the association between lncRNA DANCR expression and DFS or CPs (a, age; b, gender; c, tumor size; d, tumor differentiation; e, depth of invasion; f, lymph node metastasis; g, distant metastasis; h, clinical stage)

**Figure 6:**
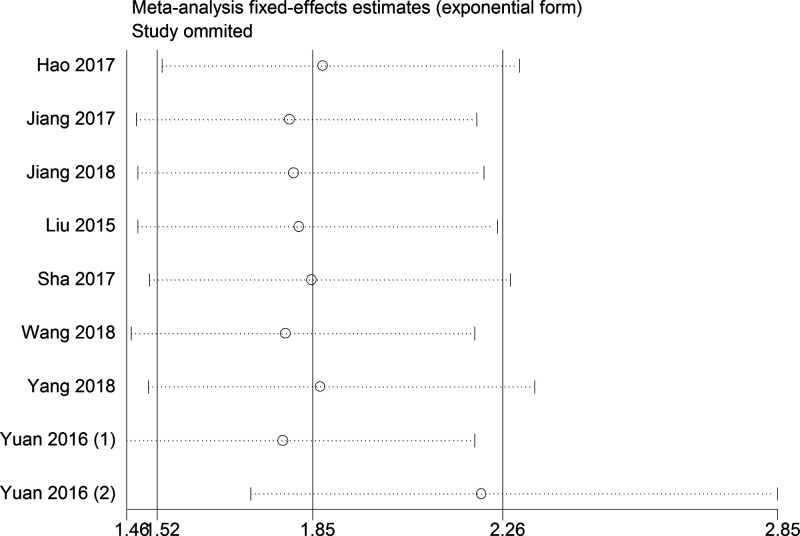
Sensitivity analysis for the meta-analysis of the association between lncRNA DANCR expression and OS

Begg’s test for the meta-analysis of OS showed that there was no obvious publication bias amongst studies (Figure 4). Funnel plots demonstrated that there was no distinct publication bias with respect to the meta-analyses of DFS and CPs (Figure 5). Sensitivity analysis indicated that the pooled results of OS were not influenced by omitting each single study (Figure 6).

## Discussion

LncRNAs have been proved to play a vital role in the tumorigenesis, differentiation, invasion, and metastasis of cancers [25,26]. Many lncRNAs have the potential capacity to predict the cancer progression and prognosis [27,28]. Recently, many studies have found that lncRNA DANCR expression might be involved with the prognosis of cancers; however, dispute remains for conflicting data amongst different studies [11–21].

In our study, we discovered that high lncRNA DANCR expression was significantly associated with shorter OS and DFS in cancers. We also found, compared with patients with low lncRNA DANCR expression, patients with high lncRNA DANCR expression tended to have deeper depth of invasion, earlier lymph node metastasis, earlier distant metastasis, and more advanced clinical stage. Unexpectedly, we failed to observe the relationship of lncRNA DANCR expression with tumor size or differentiation; however, it should be noted that the results were not reliable enough because of the distinct heterogeneity amongst included studies. Overall, high lncRNA DANCR expression was an unfavorable factor in the cancer prognosis. To our knowledge, the present study was the first meta-analysis to explore the prognostic and clinicopathological value of lncRNA DANCR expression in human cancers.

Many researches have tried to elucidate the prognostic role of lncRNA DANCR expression in cancers [11–13]; however, the underlying mechanism remains unclear. Yang et al. found that down-expression of lncRNA DANCR could increase the expression of miR-33a-5p, reduce the EMT and increase the apoptosis of glioma cells [20]. Differently, Li et al. study demonstrated that high lncRNA DANCR expression could positively affect the progression of glioma through activating the Wnt/β-catenin signaling [29]. Besides, lncRNA DANCR could mediate cisplatin resistance in glioma cells via activating the AXL/PI3K/Akt/NF-κB signaling pathway [30]. Wang et al. study revealed that lncRNA DNACR facilitated the invasion and metastasis of osteosarcoma by promoting the ROCK1-mediated progression through decoying both miR-1972 and miR-335-5p [19]. Besides, lncRNA DANCR could promote the HSP27 expression and its mediation of metastasis via miR-577 sponging in colorectal cancer [31]. Zhen et al. results showed lncRNA DANCR could promote the progression of lung cancer by sequestering the miR-216a [32]. Lu et al. study, also focussing on lung cancer, discovered that lncRNA DANCR expression regulated mTOR expression by directly binding to miR-496 [33]. In gastric cancer, Pan et al. found SALL4 could facilitate the lncRNA DANCR expression and exert its oncogenic activities via activating the β-catenin pathway [15]. As for prostate cancer, Jia et al. study revealed that lncRNA DANCR promoted the tumor invasion and metastasis through the down-expression of TIMP2/3 [34].

Several limitations should be considered when interpreting our results. First, only 11 studies were included into this meta-analysis, which might reduce the stringency of results. Second, most studies included were conducted in China which might result in regional bias. Third, HR and 95% CI were extracted from survival curves in several studies as described by Tierney et al. [23], which might be affected by the subjective factors of operators; however, this method has been widely accepted and used in meta-analyses [35–37]. Fourth, the prognostic value of lncRNA DANCR expression in specific cancer was not determined in the present study because of limited included studies. With a view to these limitations, prospective studies with larger population and longer follow-up time are warranted to clarify this issue.

## Conclusion

High lncRNA DANCR expression was associated with shorter OS, shorter DFS, and worse clinicopathological features compared with low lncRNA DANCR expression in human cancers. LncRNA DANCR expression could serve as a promising prognostic factor of human cancers.

## Supplementary Material

Supplementary Table S1

## Abbreviations

CP: clinicopathological parameter
DANCR: differentiation antagonizing non-protein coding RNA
DFS: disease-free survival
HR: hazard ratio
NOS: Newcastle–Ottawa Scale
OR: odds ratio
OS: overall survival
qRT-PCR: quantitative reverse transcription PCR
RFS: recurrence-free survival
